# Pretreatment volume-based ^18^F-FDG PET/CT parameters as prognostic indicators in malignant peritoneal mesothelioma patients

**DOI:** 10.1007/s11604-026-01977-9

**Published:** 2026-03-21

**Authors:** Kazuhiro Kitajima, Kosuke Matsuda, Hiroyuki Yokoyama, Toshiyuki Minami, Akifumi Nakamura, Kozo Kuribayashi, Takashi Kijima, Koichiro Yamakado

**Affiliations:** 1 Department of Radiology, Hyogo Medical University, 1-1 Mukogawa-cho, Nishinomiya, Hyogo 663-8501 Japan; 2 Department of Respiratory Medicine and Hematology, Hyogo Medical University, Nishinomiya, Hyogo Japan; 3 Department of Thoracic Surgery, Hyogo Medical University, Nishinomiya, Hyogo Japan

**Keywords:** Malignant peritoneal mesothelioma (MPeM), Survival, ^18^F-fluorodeoxyglucose (FDG), Positron emission tomography/computed tomography (PET/CT), Maximum standardized uptake value (SUV), Metabolic tumor volume (MTV)

## Abstract

**Objective:**

This study was conducted to examine relationships of pretreatment volume-based quantitative ^18^F-fluorodeoxyglucose (^18^F-FDG) positron emission tomography/computed tomography (PET/CT) parameters with overall survival (OS) in malignant peritoneal mesothelioma (MPeM) patients.

**Materials and methods:**

Data for 71 patients with FDG-avid MPeM who underwent pretreatment ^18^F-FDG PET/CT were retrospectively reviewed. The highest maximum standardized uptake value (SUVmax), metabolic tumor volume (WB MTV), and total lesion glycolysis (WB TLG) were calculated, including primary tumors and metastatic lesions. Relationships of clinicopathological factors (histological subtype, primary peritoneal disease form, abdominal nodal metastasis, extra-abdominal metastasis, treatment regimen), as well as volume-based quantitative PET/CT parameters with OS were evaluated using a Cox proportional hazards model and log-rank test.

**Results:**

Enrolled patients underwent follow-up for a mean period of 27.6 months (range 2.1–161.2 months, median 18.9 months), during which 49 (69.0%) died. Receiver operating characteristic curve analysis and log-rank testing indicated that those with high SUVmax (≥ 6.9), WB MTV (≥ 60), or WB TLG (≥ 230) had a significantly lower OS rate than patients with a low rate (< 6.9, < 60, < 230; p = 0.0002, p < 0.0001, p < 0.0001, respectively). Univariate analysis of all patients indicated an association of diffuse peritoneal disease form (p = 0.022), high level SUVmax (p = 0.0002), WB MTV (p < 0.0001), or WB TLG (p < 0.0001) level, extra-abdominal metastasis (p = 0.079) and treatment regimen (p = 0.058) with significantly shorter OS. Additionally, multivariate analysis results confirmed high WB MTV as an independent negative predictor (hazard ratio 2.51, 95% confidence interval 0.72–13.45; p = 0.039).

**Conclusions:**

These findings indicate that pretreatment volume-based quantitative ^18^F-FDG PET/CT parameters, especially whole-body MTV, may be useful as surrogate markers for MPeM prognosis.

## Introduction

Malignant peritoneal mesothelioma (MPeM), most commonly caused by exposure to asbestos, is a rare but aggressive type of cancer arising from mesothelial cells of the peritoneum that accounts for approximately 7–30% of all diagnosed mesothelioma cases [1, 2]. This cancer typically exhibits rapid, diffuse, and extensive spreading throughout the abdomen, with most affected patients dying from the disease within one year. Signs and symptoms of MPeM are generally non-specific, and include abdominal pain and distension as well as weight loss, though diagnosis is often delayed because of its rarity and nonspecific presentation. A previous report noted a median interval of four to six months from initial presentation to diagnosis [3]. Biopsy results along with histological and immunohistochemical findings are required for definitive diagnosis of this challenging disease.

For non-invasive prediction of tumor biological aggressiveness and factors related to patient outcome, positron emission tomography/computed tomography (PET/CT) using ^18^F-fluorodeoxyglucose (^18^F-FDG) to determine increased glucose uptake by malignant cells has been advocated. Maximum standardized uptake value (SUVmax), a semi-quantitative parameter derived from PET, is currently the most commonly used indicator for cancer examinations, however, it represents only the single point of highest metabolic activity within a tumor and does not reflect the overall metabolic tumor burden. Thus, metabolic tumor volume (MTV) and total lesion glycolysis (TLG) have been employed in recent years as indicators of metabolic activity throughout the entire tumor volume [4, 5], and might potentially be used to more precisely reflect tumor biology, prognosis, and response to treatment as compared to SUVmax. To the best of our knowledge, the usefulness of volume-based quantitative parameters with ^18^F-FDG PET/CT, and the utility of MTV or TLG for assessing tumor biological aggressiveness for predicting outcomes of MPeM patients has not been reported. The present study was conducted with an MPeM patient cohort to identify whether volume-based quantitative ^18^F-FDG PET/CT parameters can be used to predict overall survival.

## Materials and methods

### Patients

This retrospective study received approval from the institutional review board of our hospital (no. 3219) and the requirement for informed consent was waived. The records of 80 newly diagnosed MPeM patients who underwent whole-body ^18^F-FDG PET/CT for initial staging prior to undergoing treatment between June 2007 and October 2024 were analyzed. Patients with non-^18^F-FDG-avid MPeM lesions (n = 5) or lost to follow-up (n = 4) were excluded, thus the final study population included 71 (45 men, 26 women; median age 68 years, range 23–83 years). Results obtained in those examinations were examined by a panel of respiratory and internal medicine physicians, thoracic surgeons, radiation oncologists, and radiologists, each a member of the Thoracic Tumor Board Conference of the Hyogo College of Medicine Hospital (Hyogo, Japan), to determine clinical staging and treatment. Demographic data and clinicopathological parameters, including histological subtype, treatment strategies, and OS, were obtained, with patient and tumor characteristics presented in Table 1.

**Table 1:** Patient characteristics

Character	N	%
*Sex*		
Male	45	63.4
Female	26	36.6
*Age*		
Mean	63.8 ± 13.7	
Range	23–83	
*Diagnostic tool*		
Laparoscopic peritoneal biopsy	65	91.5
Ultrasound-guided core-needle biopsy	3	4.2
Biopsy during the surgery of other disease	3	4.2
*Histological subtypes*		
Epithelial	62	87.3
Biphasic	5	7.0
Sarcomatoid	4	5.6
*Abdominal nodal metastasis*		
No	65	91.5
Yes	6	8.5
*Extra abdominal metastasis*		
No	55	77.5
Yes	16	22.5
*First treatment regimen*		
CDDP + PEM	60	84.5
Nnivolumab	11	15.5

CDDP: cisplatin, PEM: pemetrexed

Sixty patients received chemotherapy with cisplatin and pemetrexed as first-line treatment, while 11 underwent treatement with nivolumab. To detect disease recurrence and/or metastasis, follow-up evaluations included physical examinations as well as ^18^F-FDG PET/CT, CT, or brain magnetic resonance imaging were performed. When recurrence or progression was suspected, the patient was treated clinically as appropriate.

### ^18^F-FDG PET/CT

The ^18^F-FDG PET/CT examinations were performed using four different PET/CT scanners installed at our institution (Gemini GXL16, Gemini TF64, Ingenuity TF: Philips Medical Systems, Eindhoven, The Netherlands; Discovery IQ: GE Healthcare, Waukesha, WI, USA). Prior to the examination, each patient was instructed to fast for five hours and blood glucose level was determined immediately prior to ^18^F-FDG injection (4.0 MBq/kg body weight for GXL16, 3.0 MBq/kg for TF64, 3.7 MBq/kg body weight for Ingenuity TF and Discovery IQ), with all in the present cohort showing a level lower than 160 mg/dL. Approximately 60 min following the injection, static emission images were acquired. For attenuation correction and anatomic localization, helical CT scan imaging was performed from the top of the head to mid-thigh using the following settings: tube voltage 120 kV (all four scanners); effective tube current auto-mA up to 120 mA for GXL16, 100 mA for TF64, 155 mA for Ingenuity TF, and 15–390 mA (Smart mA, noise index 25) for Discovery IQ; gantry rotation speed 0.5 s; detector configuration 16 × 1.5 mm for GXL16, 64 × 0.625 mm for TF64 and Ingenuity TF, and 161.25 mm or Discovery IQ; slice thickness 2 mm; and transverse field of view of 600 mm for GXL16, TF64, and Ingenuity TF, and 700 mm for Discovery IQ. Immediately following the CT examination, PET imaging was performed from the head to mid-thigh for 90 s (GXL16, TF64, Ingenuity TF) or 180 s (Discovery IQ) for each bed position in three-dimensional mode. During PET scanning, the patient was asked to breathe normally. For the GXL16, attenuation-corrected PET images were reconstructed using a line-of-response row-action maximum likelihood algorithm, and for the TF64 and Ingenuity an ordered subset expectation maximization (OSEM) iterative reconstruction algorithm was used (33 subsets, three iterations), while Q.Clear with a block sequential regularized expectation maximization (BSREM) value of 400 was utilized for the Discovery IQ.

### Imaging analysis

**Fig. 1:**
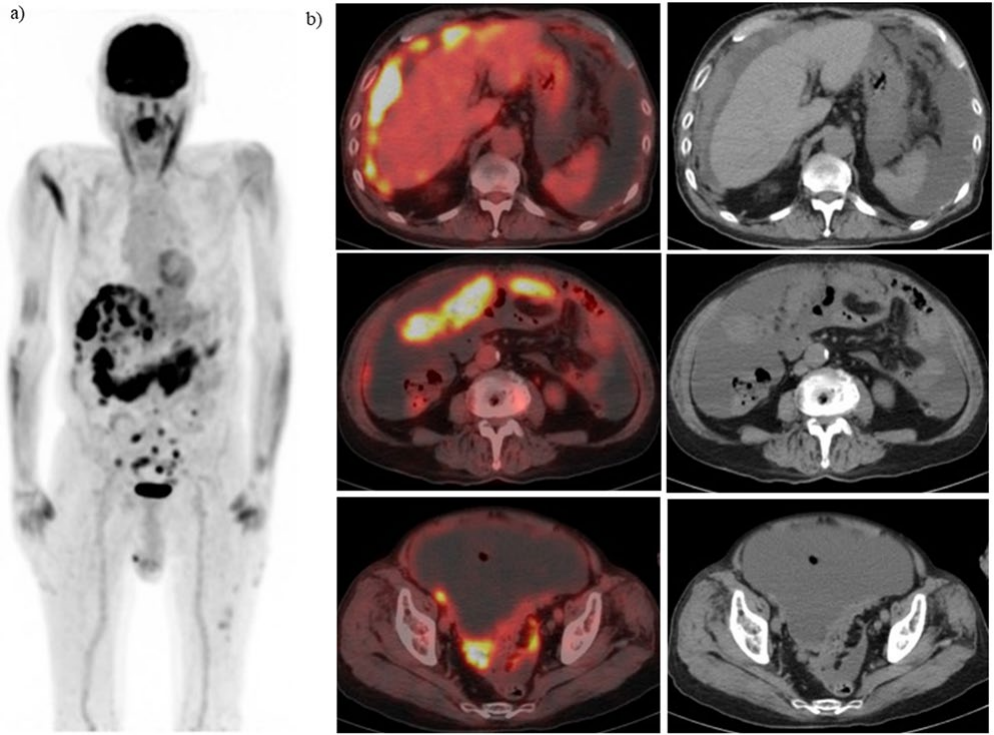
Representative findings of an 80-year-old male with epithelioid malignant peritoneal mesothelioma (diffuse type). **a** Maximum intensity projection (MIP) of ^18^F-fluorodeglucose positron emission tomography/computed tomography (^18^F-FDG-PET/CT) showing diffuse strong FDG uptake in the abdomen and pelvis. **b** Axial fused FDG-PET/CT and CT images showing diffuse, tumor-like peritoneal thickening with strong ^18^F-FDG uptake in the peritoneum and omentum. Highest maximum standardized uptake value (SUVmax) was 15.38, whole-body metabolic tumor volume (WB MBV) was 602.56 mL, and whole-body total lesion glycolysis (WB TLG) was 2510.73. Radiological peritoneal cancer index (PCI) was score 14. Cisplatin and pemetrexed were administered, however, the patient died 113 days after diagnosis

Each of the ^18^F-FDG PET/CT images was retrospectively reviewed by an experienced reader with 15 years of experience with oncologic ^18^F- FDG PET/CT examinations and no knowledge of other imaging results, or access to clinical or histopathologic data for the patients. The commercially available software package RAVAT (Nihon Medi-Physics Co. Ltd., Tokyo, Japan) was used, which is able to harmonize SUVs obtained with different PET/CT systems in a range advocated by the Japanese Society of Nuclear Medicine (JSNM) using phantom data [6]. SUVmax was defined as maximum SUV within the target volume and determined using the following formula: concentration of radioactivity in volume of interest (VOI) (MBq/ml) × total body weight (kg)/injected radioactivity (g/MBq). SUVmean values were calculated as the summed SUV of all voxels in the target volume divided by number of voxels in that volume. MTV was automatically determined inside the tumor VOI using a margin threshold set at 40% of SUVmax. Then, with both metabolic activity and tumor burden taken into consideration, TLG was calculated as SUVmean × MTV. Highest SUVmax was defined as the maximum value for all primary tumor and metastatic lesions, and whole-body MTV (WB MTV) and whole-body TLG (WB TLG) were calculated by summing the corresponding values for each lesion in the patient. Additionally, radiological peritoneal cancer index (PCI) of each case was retrospectively evaluated. Findings of a representative case are presented in Fig. 1.

### Statistical analysis

Continuous data are presented as median and range, and categorical data as number and percentage. OS was defined as the time interval in months between the date of the initial ^18^F-FDG PET/CT scan and date of censor for survivors or of death. Receiver operating characteristic (ROC) curves and Youden’s Index were utilized to confirm cut-off point validity for SUVs, MTV, and TLG. Optimal cut-off point for radiological PCI was estimated using median value. The Kaplan–Meier method was used to calculate mean OS, which was confirmed with a log-rank test. To identify independent predictors with influence on prognosis, a Cox proportional hazards model was used to evaluate the effects of multiple risk factors considered to have a significant impact on OS. Variables with a P value of < 0.1 shown in univariate analysis were subjected to multivariate analysis. SAS, version 9.3 (SAS Institute Inc., Cary, NC, USA), was used to conduct all statistical analyses, with two-tailed p values < 0.05 considered to indicate statistical significance.

## Results

**Fig. 2:**
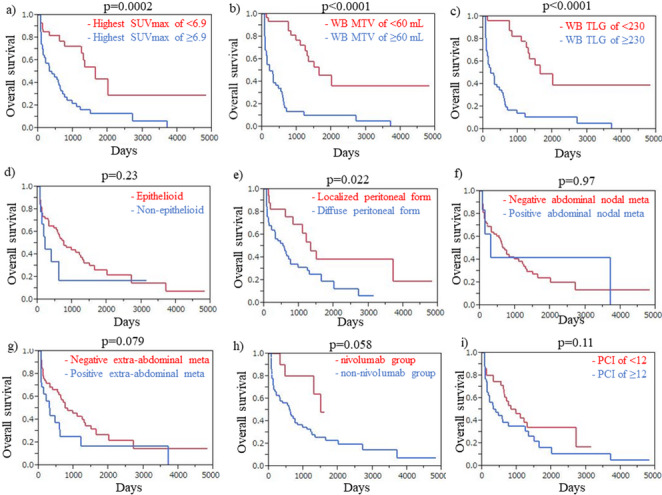
Kaplan–Meier overall survival curves for 71 patients with malignant peritoneal mesothelioma stratified based on **a** non-epithelioid histological subtype, **b** diffuse primary peritoneal disease form, **c** positive for abdominal nodal metastasis, **d** positive for extra-abdominal metastasis, **e** non-nivolumab treatment, **f** highest SUVmax ≥ 6.9, **g** highest WB MTV ≥ 60 mL, **h** highest WB TLG ≥ 230, and **i** PCI ≥ 12

The 71 enrolled patients underwent follow-up examinations for a mean period of 27.6 months (range 2.1–161.2 months, median 18.9 months), during which 49 (69.0%) died. For all enrolled patients, the median values for highest SUVmax, WB MTV, and WB TLG were 7.36 (range 2.37–22.03), 113.33 mL (range 2.01–3273.09 mL), and 674.14 g (range 4.98–14,487.21 g), respectively, while the mean ± standard deviation values for those were 7.78 ± 3.82, 415.11 ± 642.93 mL, and 1902.89 ± 2945.18 g, respectively. ROC curve analysis and log-rank testing showed that patients with a high SUVmax value (≥ 6.9) had a significantly lower OS rate as compared to those with a low SUVmax value (< 6.9) (p = 0.0002) (Fig. 2a), while patients with a high WB MTV value (≥ 60) had a significantly lower OS rate than those with a low WB MTV value (< 60, p < 0.0001) (Fig. 2b). Using the ROC curve analysis and log-rank results, the patients were divided into two groups according to WB TLG (< 230 vs. ≥ 230, p < 0.0001) (Fig. 2c).

Next, univariate analysis of factors with potential influence on OS (histological subtype, primary peritoneal disease form, abdominal nodal state, extra abdominal metastasis state, treatment regimen, highest SUVmax, WB MTV, WB TLG) was performed. Not associated with significantly shorter OS were non-epithelioid histological subtype (p = 0.23), abdominal nodal metastasis (p = 0.97), whereas diffuse peritoneal disease form (p = 0.022), extra-abdominal metastasis (p = 0.079) and treatment regimen (p = 0.058) as well as high highest SUVmax (p = 0.0002), WB MTV (p < 0.0001), and WB TLG (p < 0.0001) each showed an association (Table 2, Fig. 2a-h). Furthermore, multivariate analysis results confirmed high WB MTV (hazard ratio 2.51, 95% confidence interval 0.72–13.45; p = 0.039) as an independent predicter of shorter OS (Table 2). Extra-abdominal metastasis, non-nivolumab treatment, diffuse peritoneal disease form, high highest SUVmax, and high WB TLG were not independent predictors of shorter OS (Table 2).

**Table 2:** Factors associated with OS with all 71 patients

Item	Number of patients	MST (m)	Univariate analysis		Multivariate analysis	
			p (log-rank)	Hazard ratio (95% CI)	p (log-rank)	Hazard ratio (95% CI)
*Histology*						
Epithelioid	62	20.3	0.23	1.63 (0.66–3.45)		
Non-Epithelioid	9	6.7				
*Primary peritoneal disease form*						
Localized	17	36.8	0.022	2.30 (1.16–5.10)	0.16	2.36 (1.04–5.90)
Diffuse	54	15.2				
*Abdominal nodal metastasis*						
No	65	19.6	0.97	0.98 (0.29–2.49)		
Yes	6	7.1				
*Extra abdominal metastasis*						
No	55	21.0	0.079	1.74 (0.90–3.18)	0.56	1.26 (0.57–2.63)
Yes	16	10.3				
*Highest SUVmax*						
< 6.9	28	35.0	0.0002	3.23 (1.72–6.51)	0.59	1.29 (0.53–3.36)
≧ 6.9	43	10.8				
*WB MTV*						
< 60	30	41.1	< 0.0001	5.64 (2.97–11.37)	0.039	2.51 (0.72–13.45)
≧ 60	41	6.7				
*WB TLG*						
< 230	28	42.3	< 0.0001	6.03 (3.10–12.72)	0.51	1.75 (0.29–7.62)
≧ 230	43	6.7				
*Radiological PCI*						
≦ 11	41	19.1	0.11	1.57 (1.03–3.12)		
≧ 12	30	11.6				
*First treatment regimen*						
Nivolumab	11	22.2	0.058	2.61 (1.05–8.70)	0.23	1.84 (0.71–6.31)
CDDP + PEM	60	17.6				

OS: overall survival, MST: median survival time, CI: confidence interval, SUVmax: standardized uptake value, WB MTV: whole-body metabolic tumor volume, WB TLG: whole-body total lesion glycolysis, PCI: peritoneal cancer index, CDDP: cisplatin, PEM: pemetrexed

Radiological PCI was of 71 patients were score 4 for six patients, score 5 for two patients, score 6 for nine patients, score 7 for four patients, score 8 for eight patients, score 9 for two patients, score 10 for four patients, score 11 for six patients, score 12 for six patients, score 13 for two patients, score 14 for two patients, score 16 for two patients, score 18 for five patients, score 19 for one patient, score 20 for five patients, score 21 for one patient, score 22 for two patients, score 23 for one patient, score 28 for two patients, and score 32 for one patient. The median radiological PCI was 11. ROC curve analysis and log-rank testing showed that patients with a high PCI (≥ 12) had an insignificantly lower OS rate as compared to those with a low PCI (≤ 11) (p = 0.11) (Table 2, Fig. 2i).

## Discussion

There were two major findings obtained in the present study. First, volume-based quantitative parameters, especially whole-body MTV, shown by ^18^F-FDG PET/CT findings prior to treatment are significant predictors of OS in patients with MPeM and could serve as potential surrogate markers for prognosis. Although highest SUVmax and whole-body TBU were also significantly associated with poor survival, whole-body MTV showed the strongest association among those three quantitative parameters in the present series of patients. Notably, the diffuse peritoneal disease form was also a negative predictor of OS. Accordingly, MPeM patients who exhibit diffuse spreading of a high volume of primary peritoneal disease are speculated have the poorest survival. As for the other major finding, histological subtype and metastatic lesions were shown to be not associated with poor survival. Contrary to our expectation, MPeM patients with a biphasic or sarcomatoid histology subtype, and those with nodal or distant metastasis showed generally good survival. However, such cases were relatively few in the present series, thus it will be necessary to evaluate additional cases.

Histologically, MPeM is classified into epithelioid, sarcomatoid, and biphasic (mixed) subtypes, with epithelioid the most common subtype, noted in approximately 75% of affected patients, and generally associated with the best prognosis. It has been reported that approximately 25% of MPeM patients are biphasic, while the sarcomatoid subtype is exceedingly rare, though both of these subtypes are associated with significantly worse prognosis, similar to corresponding pleural mesothelioma variants [1]. An epithelial mesothelioma does not typically invade solid organs, while omentum infiltration is detected in most affected cases [7]. The disease usually remains confined to the abdomen and multiple affected sites throughout the peritoneum have been reported. The sarcomatoid type is generally more infiltrative and shows more rapid growth, while the biphasic type shows radiological and gross pathological features also found in both the epithelial and sarcomatoid types. In advanced stage cases, pleural cavity involvement and distant metastases may occur.

There are two basic forms of MPeM, diffuse and localized type. The first is characterized by diffuse nodules and plaques that tend to encase the bowel viscera, while the localized form is characterized as a large tumor mass, usually in the upper abdomen, with discrete nodules scattered throughout the peritoneum [8]. Another study noted that approximately 82.1% of all cases are the diffuse type [9]. Contrast-enhanced CT is widely accepted as the primary imaging modality for management of MPeM, as it offers such advantages as excellent spatial anatomical details, rapid acquisition, and broad availability. CT findings commonly associated with MPeM include ascites, peritoneal thickening and caking, omentum thickening or masses, mesenteric nodules, small bowel involvement with solid or cystic masses, scalloping of intraabdominal organs, and pleural plaques [1]. Local–regional invasion is commonly encountered. In contrast, lymph node involvement (5–10%) and extra-abdominal metastasis (3–5%) are relatively rare complications, and generally considered to be associated with advanced late-stage disease [10, 11].

PCI which could intraoperatively visualize extent and score 13 abdominal regions according to the size and number of peritoneal deposits/lesions, has shown to be associated with OS, with increasing burden of disease associated with decreased survival [12, 13]. The value of radiologic-PCI has previously been reported in the literature [14]. Our series showed PCI was a weak negative OS predictor in MPeM patients without significant difference, proved to be inferior surrogate marker than WB MTV.

The present study was limited by its retrospective design and the possibility of selection bias. Additionally, the number of patients was relatively small. Nevertheless, given the rarity of MPeM, such limitations are inevitable. In addition, the follow-up period of the nivolumab group was relatively short, because nivolumab treatment has been introduced relatively recently.

## Conclusion

Pretreatment volume-based quantitative ^18^F-FDG PET/CT parameters, especially whole-body TLG, appear to be promising surrogate markers for prognostication in MPeM.
